# Palliative Care and End-of-Life Issues in Elderly Cancer Patients With Head and Neck Cancer

**DOI:** 10.3389/fonc.2022.769003

**Published:** 2022-03-04

**Authors:** Dirk Schrijvers, Rodger Charlton

**Affiliations:** ^1^Department of Medical Oncology, Ziekenhuisnetwerk Antwerpen, Antwerp, Belgium; ^2^Leicester Medical School, College of Life Sciences, University of Leicester, Leicester, United Kingdom

**Keywords:** geriatric palliative care, head and neck cancer, pain, malnutrition, end-of life care, fatigue, locoregional problems

## Abstract

The number of elderly patients with incurable head and neck cancer will increase. They are in need of geriatric palliative care, that takes into account oncology, palliative care and geriatric medicine. In this review of the most recent and relevant literature and includes the expert opinion of the authors, several physical problems (e.g. pain, fatigue, malnutrition, and loco-regional problems) encountered by the elderly head and neck cancer patients are addressed. In addition end-of life issues in this patient population are discussed.

## Introduction

The number of head and neck cancers is high in elderly patients and the peak incidence of head and neck cancer is around 70-74 years (1).

Head and neck cancer is located at different regions of the head and neck (e.g. nasopharyngeal, oropharyngeal, oral cavity, hypopharyngeal and laryngeal), each having its own prognosis, and are diagnosed at different stages, each necessitating a specific and in many cases a multimodal treatment approach. It is difficult to apply international treatment guidelines to all elderly due to the differences in global geriatric health profiles among them. In some elderly patients multi-modal treatment may be used (e.g. chemoradiation), but a German retrospective single-center analysis which enrolled all patients older than 65 years treated with radiotherapy or chemoradiation for histologically confirmed head and neck squamous cell carcinoma between 2010 and 2018, combined modality treatment seems to translate in a lower overall survival rate than in younger patients, so that single agent treatment may be considered in this patient group (2).

In the United Kingdom (UK), five-year survival rates again depend on the head and neck region involved and varies from around 20% for men and women aged 70-89 years diagnosed with hypopharyngeal cancer to 58% in 80-99 year-old patients diagnosed with laryngeal cancer (1). These figures will vary globally. In relation to head and neck squamous cell carcinoma 25% of these patients are older than 70 years at the time of diagnosis, and this percentage will further increase in Western countries due to ongoing demographic trends (2).

It can be seen therefore that a substantial number of elderly patients will not be cured of their disease or will relapse and these patients are in need of palliative care.

Palliative care is defined as an approach that improves the quality of life (Qol) of patients and their families who are facing problems associated with a progressive life-threatening illness. It prevents and relieves suffering through the early identification, correct assessment and treatment of pain and other problems, whether physical, psychosocial or spiritual (3).

Although palliative care has been implemented in the 1980s in cancer care, many patients still suffer unnecessarily: according to the World Health Organisation (WHO), an estimated 40 million people worldwide are in need of palliative care, while only about 14% of people who need palliative care currently receive it (3). Also it has been shown that integrating palliative care early in the disease trajectory translates in a better Qol of cancer patients with advanced disease (4).

In elderly patients, integration of geriatric medicine and palliative care is still a problem and specific attention should be given as to geriatric palliative care. Geriatric palliative care is a field of inter-specialty collaboration unifying competences from geriatric medicine, oncology and palliative care to respond to the socio-demographic changes and challenges of older adults with severe and life-limiting conditions (5, 6).

It is our experience that elderly patients with head and neck cancer experience problems in the physical field (e.g. pain, nutritional issues due to dysphagia and swallowing disorders, soft tissue oedema in the head and neck region, fistulas), psychological problems (e.g. depression, anxiety), social issues (e.g. isolation) and spiritual issues [e.g. end-of life (EoL) decisions, advanced care planning]; while problems related to frailty (e.g. sarcopenia, polypharmacy, falls, incontinence) may add to the problems related to the cancer. The presence of frailty in elderly patients with head and neck cancer translates in a worse prognosis in elderly patients (7).

And although elderly patients with cancer experience similar problems as younger patients, they may express their problems to a lesser or inadequate extent (8).

In this chapter, the palliative approach of some symptoms and EoL issues related to head and neck cancer in elderly patients is discussed. Since head and neck cancer in the elderly is an under-researched area, extrapolations of some issues are made from head and neck cancer and geriatric oncology.

## Physical Symptoms in Geriatric Patients With Advanced Head and Neck Cancer

Patients with advanced head and neck cancer in palliative care setting report a diverse range of physical symptoms ranging from pain, fatigue or lack of energy, difficulty eating or swallowing, dry mouth, incontinence, bleeding, dyspnoea, fungating lesions, change in appetite, cough, communication difficulties, constipation, retained mucus, soft tissue oedema, and insomnia (5, 6).

### Pain

Pain is commonly reported in patients with advanced head and neck cancer with prevalences ranging from 40% to 95%, and increases with more advanced disease states (9). It is complex in nature with nociceptive and neuropathic components. In addition to cognitive and functional impairments; and co-morbidity, the different pathogenic mechanisms may hamper adequate pain control.

The assessment of pain in elderly head and neck cancer patients should involve a comprehensive multidisciplinary evaluation with a complete physical examination and structured pain review with the use of validated scales in patients able to communicate [e.g. visual analogue scale, neuropathic pain diagnostic questionnaire (DN4), neuropathic pain scale] or in patients with an impaired cognition (e.g. Pain Assessment Checklist for Seniors with Limited Ability to Communicate; Doloplus-2 Scale; Rotterdam Elderly Pain Observation Scale; Elderly Pain Caring Assessment-2).

Elderly patients with head and neck cancer prefer a numerical scale to score pain intensity (10).

Treatment should be according to the guidelines provided by the WHO analgesic ladder and should be specifically adapted to the type of pain (11).

Non-pharmacological Interventions should be combined with pharmacological interventions and can be provided by a multidisciplinary team including a geriatrician, palliative care specialist, psychologist, social and spiritual worker.

Non-pharmaceutical interventions exits of physiotherapy, massage, relaxation techniques, exercise, and rehabilitation. Cognitive behavioural therapy may be helpful if the non-cognitive impaired patient. However, their impact on pain in elderly patients with head and neck cancer is not well studied.

Pharmacological interventions in elderly patients should take into account age-related changes in metabolism (e.g. kidney and liver dysfunction, nutritional status, sarcopenia) and drug-drug interactions.

Non-opioids (e.g. paracetamol) are used to treat mild to moderate nociceptive pain in the elderly cancer patients. The patient should be counselled in relation to the daily maximum dose taking into account liver function disturbances. Nonsteroidal anti-inflammatory drugs (NSAIDs) are also indicated for the treatment of mild to moderate nociceptive pain, especially processes where inflammation is prominent. NSAIDs are associated with increased complication risks in the older patient such as gastrointestinal bleeds, renal toxicity, myocardial infarction, and stroke.

Opioids are used in the treatment of moderate to severe cancer-related pain. Before initiating treatment it is important to evaluate the organ function, cognitive status, social support, and potential drug-drug interactions with other medications. In the older cancer patient, oral administration is preferred because of ease of use and affordability.

Weak opioids (e.g. codeine, hydrocodone, tramadol, buprenorphine) are the second step in the WHO analgesic ladder and are recommended for mild-moderate pain. Strong opioids (e.g. morphine, methadone, fentanyl, oxycodone, hydromorphone) are indicated for moderate to severe pain (step 3 of the WHO ladder), but the use of lower doses of strong opioids can make the second step of the ladder obsolete.

When starting opioids, side effects (e.g. constipation, increased xerostomia, nausea, pruritus, urinary retention, sedation, confusion, and hallucinations) should be carefully looked for, because they may be more pronounced in the elderly cancer patient. Constipation should be prevented by use of stimulant laxatives.

In case of a neuropathic pain component, classical analgesics may be less effective and adjuvant medication should be combined with them early in pain management (12). Treatment consists preferably of anti-epileptics (e.g. gabapentin, pregabalin), since anti-depressant drugs, another group of medication active in neuropathic pain are less indicated in elderly because of significant anticholinergic side effects and possible cognitive changes.

Anti-epileptics should be escalated slowly in the older cancer patient and should take into account renal impairment.

Serotonin-norepinephrine reuptake inhibitors (e.g. venlafaxine, duloxetine) may also be used to treat neuropathic pain and are well tolerated (13).

### Fatigue and Lack of Energy

Fatigue or lack of energy is frequently reported in elderly patients with cancer and prevalences range from 77% to 81% (5).

Cancer-related fatigue (CRF) is characterized by feelings of tiredness, weakness, and lack of energy, and is not relieved by rest or sleep.

Different validated scales have been developed to evaluate CRF (e.g. Functional Assessment of Cancer Therapy Fatigue, European Organisation for Research and Treatment of Cancer Quality of Life Questionnaire (EORTC QLQ C30) (fatigue subscale), Fatigue Questionnaire), that may be used to evaluated the severity of CFR, the evolution of CFR and the effect of an intervention (14).

CRF is associated with distress, depression, anxiety, and low performance status as well as other symptoms such as nausea, vomiting, lack of appetite, sleep disturbance, dyspnoea, dry mouth, restlessness, and problems with concentration (13).

There is no ideal solution for CRF since it is multifactorial in cause. In case of an identifiably cause (e.g. hypothyroidism, anaemia) a specific treatment can be started.

Non-pharmacological interventions exist of life style adaptations (e.g. energy conservation measures, regular rest periods, selection of exercise expenditure); mild aerobic and resistance exercise regimens and psychological interventions (e.g. stress management, relaxation). These interventions have not been properly evaluated in the palliative care setting of elderly patients with head and neck cancer.

Pharmacological interventions rarely provide a long-term improvement of fatigue, but corticosteroids may lead to a temporary relief of fatigue complaints (15).

### Malnutrition/Cachexia/Dysphagia

Patients with head and neck cancer may develop malnutrition due to the disease itself but also due to prior treatments.

Appetite loss, anorexia, taste and smell changes, nausea and vomiting, oral mucositis, pain, oropharyngeal (recurrent) disease, oropharyngeal dysphagia and constipation may result in malnutrition leading to cachexia (16).

Malnutrition is the cause of death in 20–30% in cancer patients (17).

Oropharyngeal dysphagia (OD) is a common complication due to head and neck cancer and its treatment. It is seen in around 50-70% of patients treated with (chemo)radiation and in 10- 72% after total laryngectomy. OD may hamper normal eating and lead to aspiration pneumonia, malnutrition, dehydration, and choking. It may also lead to tube feeding dependency (15).

The causes of malnutrition should be evaluated and a cause-specific treatment in combination with a symptomatic treatment should be started (16).

In advanced cancer patients, preserving nutritional status may be a relevant topic even during the palliative care phase since nutritional deficits impair performance status, Qol, and survival.

The goal of nutritional support is to preserve oral nutrition by minimizing food-related discomfort and maximizing food enjoyment through strategies including dietary counselling by a dietician, food fortification, and oral nutritional supplements (16).

A multi-disciplinary team approach is recommended to help patients with dysphagia and so assistance to improve their swallowing with appropriate techniques, exercises and positioning through a speech and language therapist. This will include modifying the texture of food to reduce the risk of choking and at the same time ensure adequate hydration and nutrition.

Around 5-10% of patients with head and neck cancer are dependent on permanent enteral tube feeding due to prior treatments (18). In patients with head and neck cancer developing swallowing difficulties during the palliative care phase, tube feeding may be considered to maintain or improve the general condition (16). Total parenteral nutrition in patient with a life expectancy of 3 months without an active anticancer treatment is not indicated according to expert opinion (19).

If expected survival is less than 6 weeks, focussing on anti-cachexia interventions aimed at alleviating distressing symptoms like thirst, nausea, vomiting and dysphagia, and psychological and existential distress, as well as distress to family members, is recommended (19). This palliative care approach at the EoL is reviewed very regularly to maintain Qol where possible, which is proactive to anticipate symptoms and to ensure when suffering is observed, that it is alleviated appropriately and quickly.

### Loco-Regional Problems

Patients with head and neck cancer can develop loco-regional problems (e.g. facial and tongue oedema, necrotic local tumourous, which may impair severely the QoL.

#### Lymphoedema

Lymphoedema is a not well studied complication in patients with head and neck cancer, but can have a tremendous impact of the QoL of the patient.

Chronic lymphoedema is seen in a high proportion of patients with head and neck cancer after (chemo)radiation and this is reported in up to 98% of patients with more or less severity (20).

Facial oedema can be so extensive that vision and eating can become a problem, while oedema of the tongue can result in breathing and eating problems, ulcerations of the swollen tongue, and sialorrhoea.

The problem of lymphoedema should be addressed by the multidisciplinary team, including a physical therapist/certified lymphoedema therapist (21).

Manual lymph drainage (MLD) is the standard treatment of facial lymphoedema (22). Oedema of the tongue is a difficult complaint to palliate. Beside prevention of ulceration, no effective treatment has been described.

#### Necrotic/Fungating Tumoural Lesions

Ulcerating tumours in the head and neck region are observed in patients with recurrent head and neck cancer after treatment. They can cause pain, exudation and can produce a putrid odour due to anaerobic infections. They impair QoL and may cause isolation due to avoidance by the family and medical caregivers due to these complications.

Metronidazole ointment (0.75-3%) in combination with an absorbing dressing can be used to alleviate the malodour in an effective way (23).

## End-of Life Issues in the Elderly

As palliative care has developed, many different terms have been used over the last 50 years since the start of the modern hospice movement and those include terminal care, hospice care, palliative care and most recently EoL care. Many are ‘frail elderly’ patients particularly with an increasing elderly population as people are living longer and experiencing multiple co-morbidities towards the EoL.

The European Association for Palliative Care writes as their definition (24);

*“Palliative care is the active, total care of patients whose disease is not responsive to curative treatment. Palliative care takes a holistic approach, addressing physical, psychosocial and spiritual care, including the treatment of pain and other symptoms. Palliative care is interdisciplinary in its approach and encompasses the care of the patient and their family and should be available in any location including hospital, hospice and community.*

*Palliative care affirms life and regards dying as a normal process; it neither hastens nor postpones death and sets out to preserve the best possible quality of life until death.”*

## Care Planning

Timely identification of these patients is vital in the planning and provision of appropriate EoL care. The transition to a dying phase in non-acute situations is such that it is often unclear when the EoL/palliative care starts (25). It can be helpful to put patients who a family physician working in the community believes are dying from cancer onto a palliative care register so that they can be pro-actively and regularly recalled and reviewed through the provision of anticipatory care so that appropriate services can be put into place. Ascertaining how long a patient has left to live is a hugely difficult question and is one often asked by relatives. It can help to consider the question;

*‘Would you be surprised if this patient were to die in the next 6-12 months?’* (25)

This very simple question may help guide a doctor, the patient and their relatives. However, it has been said that this surprise question performs poorly to modestly as a predictive tool for death, with worse performance in non-cancer illness (26).

Using a palliative care register enables a doctor to identify and help people live well until they die, reiterating the words of Dame Cicely Saunders, the founder of the modern hospice movement. This allows preparations to be made and anticipatory care and so proactive rather than reactive care (27).

In the UK, many patients may now also complete a ReSPECT form (ReSPECT is also an acronym standing for a Recommended Summary Plan for Emergency Care and Treatment process which supports conversations about palliative care and in case of a future emergency) (28). The ReSPECT process creates personalised recommendations for a person’s clinical care and treatment in a future emergency in which they are unable to make or express choices. ReSPECT is a patient held document of personalised recommendations for a person’s future clinical care, completed following an Advance Care Planning conversation between a patient and a healthcare professional. In relation to emergencies it includes a Do Not Attempt Resuscitate Form (DNAR) if the patient and their relatives feel this is appropriate.

## Concept of a ‘Good Death’

Caring for a dying patient is difficult and has many challenges, not least that it can make a practitioner reflect on their own mortality and present a conflict in their own thinking as to how a death can be ‘good’. Nevertheless, General Practitioners (GPs) in the community, also called Primary Care Physicians and Family Physicians, which they are now referred to as, are involved in the care of patients from birth through to death and have an important position to co-ordinate palliative care and facilitate a ‘good death’ in those who are dying.

Palliative care is more than the science of symptom control and should be holistic care. This requires a doctor to work in partnership with a dying patient and meet the many challenges to enable a good death. These include provision of a listening ear, honesty and recognition and acknowledgement of the process of anticipatory grief and the isolation to which it can lead. It should always be remembered, however, prior to the palliative care phase that “although cure remains a high priority at all ages, survival was viewed as less important by patients with increasing decades of age” (29).

It is important as doctors that we strive to provide continuity of care, to be the patient’s advocate, to ensure adequate pain control and to seek the appropriate input of other team members. The greatest challenges are being available to a patient and reviewing them regularly without prior request, to anticipate and prevent crises and keep them at home until almost their last days of life if practically possible. Provision of care should be accompanied by hope. The patient must be informed that the purpose of palliative care is to alleviate suffering, ensure continuing Qol and enable a ‘good death’ when the time comes (30).

## A Key Dilemma

Research suggests that most people would prefer to die in their own homes if that were practically possible. However, there is no ‘right’ place to die in order to achieve a good death. In the UK only about 30% are able to die in their own homes. In fact, it might be said that it matters less where we die than how we die. In other cultures not all people may want to die at home.

Support of the informal carers (family and friends) is vital, yet despite the best of intentions they are often neglected by the professional caregivers. They need to be able to grieve before the death of a loved one, have the option for respite care to avoid crises and not feel guilty that they have ‘failed’ their loved one in any way. The ReSPECT form states where a patient would want to die if possible and practical. The key dilemma is attaining this for the patient.

## Recognising the Point at Which Curative Treatment Has Become Futile

A big role of the family physician is as the patient’s advocate and ensuring palliative care is available and not having futile care aimed at cure where this is not possible and creating suffering.

Facilitating a good death requires the recognition of the transition point from curative to palliative care. But there is a dilemma, the most difficult question is defining when the point of transition from curative to palliative care occurs. Furthermore, the simplistic notion of a transition turns out to be complex, as palliative care to relieve suffering and curative care may be provided at the same time. Indeed some treatments used for cure, such as chemotherapy and radiotherapy, may also be used in palliation. It may therefore be difficult to define a point where curative treatment stops and palliation begins, and a patient may opt for both.

However, the point at which further anticancer treatment becomes futile needs to be recognized by the patient and the clinician, as further treatment aimed at a cure may then not only be useless but in itself cause suffering, e.g. with chemotherapy. In other words, if treatment is continued where there is no prospect of success, there would be the unnecessary prolongation of poor quality life. A management plan needs to be negotiated and informed consent gained for continued treatment or agreement that this point has been reached so that a smooth transition to palliative care occurs.

Palliative care should be provided by a multi-disciplinary team of doctors, nurses, therapists, social workers, clergy and volunteers and involves much more than the science of symptom control. It is not solely a role for the Specialist or Family Physician alone. It is also a time when the science of symptom control is needed during in the terminal phase which requires a multidisciplinary approach. Leveraging other members of the team, including palliative care speciaists is part of what EoL care should involve and so allow Family Physicians and Specialists to meet EoL care goals globally in elderly cancer patients with head and neck cancer (31).

The phrase used earlier to enable a patient to ‘live until they die’ is important as it is never true that ‘there is nothing more that can be done’. For a palliative care patient Qol should be maintained through adequate symptom control. Healing is both about cure and helping a person come to terms with disease. False hope should be avoided, but being positive, can give ‘a will to live’

## Anticipatory Grief

An area of theory that seems to be true over half a century later for doctors in this situation is to remember the five stages of anticipatory grief;

- Denial
- Anger
- Bargaining
- Depression
- Acceptance

described by USA psychiatrist Elisabeth Kübler-Ross (32). They really do happen after breaking bad news and a patient’s GPs care may get stuck in one of these stages and acceptance may not be reached. When teaching students they feel this is too dated to be of importance and yet is a useful model.

## Giving a Prognosis

A Patient may ask;

*“How long have I got?”*

The answer is nobody knows and invariably doctors get it wrong. Yes, you can quote five year survival rates, but patients do not stick to any ‘rules’.

It is important to allow a person to prepare for the end by making a will as discussed earlier with use of a ReSPECT form, but encourage a patient to live one day to the next and make the most of each day and suggest when new problems are encountered, that each should be considered and treated appropriately. Concentrate on the good quality life that they have at present.

## Providing Anticipatory Care in the Last Year of Life

Although a patient may not die at home, it is the family physician or GP that they will see most frequently in the last year of their life. Having broken the bad news to them of their life-threatening illness, it is important to review the patient regularly to anticipate problems rather than providing reactive care. Continuity and easy access to one GP is essential as part of ensuring a ‘good death’ and building up a good doctor-patient relationship which will be needed at the end. Palliative care is difficult, but why?

It is difficult as not only can a patient not be cured, but they may ask questions like;

*“Am I dying?”*

or

*“What will it be like”*

or say,

*“I am frightened.”*

It is difficult because it reminds you of your own mortality. Freud summarised this dilemma (33);

*“Our own death is indeed unimaginable and whenever we make the attempt to imagine it we can perceive that we really survive as spectators … at bottom no one believes in his own death, or to put the same thing in another way, in the unconscious every one of us is convinced of his own immortality.”*

No wonder then, it is difficult to talk to someone about dying.

## The Terminal Phase – The Last Few Days

So far palliative care has been about communication by the doctor. When a patient is dying and when it appears that it may happen imminently in the next few days then knowledge of symptom control will be required. Four common symptoms are pain, nausea with or without vomiting, constipation and fatigue.

This section is not intended to provide the pharmacology required as this information is readily available and is dependent on the medications available in the doctor’s locality. However, for it to be effective the following needs to be considered;

- Has the patient’s views been sought as to whether they wish to die at home in conjunction with their family and carer and the equivalent of a ReSPECT form completed as detailed earlier? Or would the patient prefer to die in a hospital or hospice?
- Are the necessary resources available of a community nurse to help with the administration of medication particularly if morphine and other medications are to be delivered *via* a syringe driver subcutaneously for a patient unable to take medication by mouth?
- Is medical advice and input available 24 hours a day with access to the patient’s medical records if the GP is not available?
- Can you as the GP make yourself as available as possible in case of crises but ideally visit regularly to anticipate crises.

There are many good websites for further information in relation to symptom control (34).

Many treatments are available for symptom relief and pain in particular and so there is no excuse for any dying patient to be in pain. Skills are needed in symptom control and to overcome the taboo ideas associated with using morphine and the false notions that in a dying patient there is ceiling dose or potential of addiction to opiates. The task of communication is important, not least in explaining the need for opiates, such morphine, when the time comes and its implication as perceived by many that it is the end.

## Bereavement

Communication does not finish with the death of the patient. It could be disputed whether or not bereavement is a medical problem. It is a significant life event and it is inevitable that all doctors will encounter relatives who are experiencing bereavement.

Medication should be avoided at the time of a patient’s death for relatives as tranquillisers or sleeping tablets postpone the outpouring of grief and can lead to dependence.

The famous playwright, Williams Shakespeare in his play, Much ado about nothing, wrote,

*“Everyone can master a grief but he that has it.”*

As to whether bereavement is a medical problem, some people will start with normal grieving and this can turn to pathological grieving through depression. For a small proportion of the population the common event and process of bereavement can effect morbidity and sometimes mortality and so has potential consequences for health.

Grief is a normal reaction to bereavement, but sometimes it can be severe and overwhelming and the sufferer may need help. A paper in 1967 also showed that in the first year after death there is a 7-fold increase in mortality of bereaved compared to non-bereaved spouses. Bereavement is therefore very much a medical problem (35).

However, for the majority of the population bereavement is a natural part of the life-cycle with few or no health sequelae and care should be taken not to over medicalise grief in the majority of people. GPs should try to identify the small proportion of the population who are at risk and manage them accordingly.

## Looking After Yourself

Caring for the dying, if done well is exhausting. Ideally, a specialist and GP follows up and cares for a patient from the time of diagnosis, through to death. This means continuity of care, a named clinician who sees the person regularly to anticipate crises and avoid unnecessary emergency hospital admissions and who is available out of hours to avert crises even if just on the end of the telephone (36).

Full details of the patient’s care must be made available (diagnosis, management plan and medication) to any doctor deputising for that patient care. To provide a good care is therefore physically, emotionally, psychologically and often spiritually demanding. So when a patient dies, such is the good relationship built up that it is also a personal bereavement for the GP. As in Roger Neighbour’s consultation model (‘The Inner consultation’) a period of ‘good housekeeping’ is required for you to reflect and discuss with colleagues and to maintain contact with the grieving relatives and that you did your best (37).

Sometimes a doctor gets invited to a patient’s funeral and it is very much an individual judgement and choice whether they attend and time dependent as a busy clinician.

## Conclusion

Care of a palliative care patient who will eventually die means that there are certain patient expectations of a GP particularly and they can be summarised as follows and summarise the chapter. The patient’s expectations are for a specialist or a GP;

- To be their doctor
- Accompany them on the journey
- To follow up, even when cure is not possible
- Availability
- Be there for them
- Communication, communication, communication
- Not Sympathy or Empathy but Compassion

It is important to reflect on each dying patient which will help with the doctor’s development and care of future patients. Also to record the anniversary of their death to make contact with the relatives as a part of a good bereavement protocol. All of this is to enable a palliative care patient who is dying with head and neck cancer to have a ‘good death’ (38).

## Author Contributions

DS did write the introduction and physical symptoms. RC did write the part on end-of-life issues. Both authors reviewed and commented the chapter. All authors contributed to the article and approved the submitted version.

## Conflict of Interest

The authors declare that the research was conducted in the absence of any commercial or financial relationships that could be construed as a potential conflict of interest.

## Publisher’s Note

All claims expressed in this article are solely those of the authors and do not necessarily represent those of their affiliated organizations, or those of the publisher, the editors and the reviewers. Any product that may be evaluated in this article, or claim that may be made by its manufacturer, is not guaranteed or endorsed by the publisher.
